# Supplementary material for: Combining Computed Tomography and Histology Leads to an Evolutionary Concept of Hepatic Alveolar Echinococcosis
Source: Pathogens. 2020 Aug 4;9(8):634. doi: 10.3390/pathogens9080634 (PMC7459611; doi:10.3390/pathogens9080634)
Supplement: Supplementary file 1 [file pathogens-09-00634-s001.zip › Table S1.docx]

**S1 Table.** Classification of patients with AE in the liver, based on the EMUC-CT

| **Patient No.** | **Sample No.** | **Sex** | **Age** | **PNM**^a^ | **Serology**^b^ | **EMUC-CT type**  **considered for this study** | **EMUC-CT type**  **existing in lesion** |
| --- | --- | --- | --- | --- | --- | --- | --- |
| 1 | 30 | F | 49 | P2N0M0 | positive | type IIIa | type IIIa |
| 2 |  | F | 21 | N/A | positive | excluded | type II |
| 3 | 18 | F | 64 | P3N0M0 | positive | type II | type II |
| 4 | 8 | M | 42 | P3N0M0 | positive | type I | type I |
| 5 |  | F | 30 | P3N0M0 | positive | excluded | type II; type IV |
| 6 | 9; 41 | F | 29 | P2N0M0 | negative | type I; type IV | type I; type IV |
| 7 | 33 | F | 63 | P3N0M0 | negative | type IV | type IV |
| 8 |  | F | 17 | P1N0M0 | negative | excluded | type II |
| 9 |  | M | 56 | P3NXMX | positive | excluded | type II |
| 10 | 11 | M | 64 | P2N0M0 | negative | type I | type I |
| 11 | 7 | M | 70 | P1N0M0 | positive | type I | type I |
| 12 | 38 | F | 48 | N/A | negative | type IV | type IV |
| 13 | 21 | F | 61 | P4N0M0 | positive | type II | type II |
| 14 |  | M | 58 | P4N1M0 | positive | excluded | type IIIb |
| 15 | 13 | F | 20 | N/A | positive | type II | type II |
| 16 |  | M | 24 | P2N0M0 | positive | excluded | type II; type IV |
| 17 | 23 | M | 35 | P4NXMX | positive | type IIIb | type IIIb |
| 18 | 45 | F | 49 | P3N0M0 | negative | type V | type V |
| 19 |  | M | 41 | P2NXM0 | positive | excluded | type II |
| 20 | 25 | F | 23 | P2N0M0 | negative | type IIIa | type IIIa |
| 21 | 1 | M | 54 | P2N1M0 | positive | type I | type I |
| 22 | 16 | F | 21 | P3N1M0 | positive | type II | type II |
| 23 | 37 | F | 68 | P2N0M0 | negative | type IV | type IV |
| 24 |  | F | 79 | P2NXMX | positive | excluded | type I |
| 25 |  | M | 70 | P4N0M0 | positive | excluded | type IIIb |
| 26 | 22 | F | 46 | P2N0M0 | negative | type II | type II |
| 27 |  | M | 23 | P2N0M0 | positive | excluded | type II |
| 28 |  | F | 71 | P2N0M0 | positive | excluded | type IIIa |
| 29 |  | M | 17 | P2N1MX | positive | excluded | type I |
| 30 | 28 | F | 25 | P2N0M0 | positive | type IIIb | type IIIb |
| 31 | 44 | F | 63 | P3N0M0 | positive | type V | type V |
| 32 | 24 | M | 28 | P2N0M0 | positive | type IIIb | type IIIb |
| 33 |  | F | 48 | P2N0M0 | positive | excluded | type IIIa |
| 34 | 27 | F | 39 | P4NXMX | positive | type IIIb | type IIIb |
| 35 | 17 | F | 20 | P2N1M0 | positive | type II | type II |
| 36 |  | F | 57 | P2N0M0 | positive | excluded | type I |
| 37 | 20 | M | 54 | P2N0M0 | positive | type II | type II |
| 38 | 14 | F | 37 | N/A | negative | type II | type II |
| 39 | 26 | F | 36 | P3N0M0 | positive | type IIIa | type IIIa |
| 40 |  | F | 44 | P3N0M0 | positive | excluded | type I |
| 41 | 10 | F | 36 | P2N0M0 | positive | type I | type I |
| 42 | 32 | F | 35 | P2N0M0 | positive | type IIIb | type IIIb |
| 43 | 6 | F | 47 | P4N0M0 | positive | type I | type I |
| 44 | 19 | F | 53 | P2N0M0 | positive | type II | type II |
| 45 | 2 | M | 22 | P2N0M0 | positive | type I | type I |
| 46 | 34 | M | 50 | N/A | negative | type IV | type IV |
| 47 | 4 | M | 46 | P4N1M0 | positive | type I | type I |
| 48 | 36 | M | 21 | P1N0M0 | negative | type IV | type IV |
| 49 | 29 | F | 54 | P2N1M0 | positive | type IIIb | type IIIb |
| 50 |  | M | 24 | P4N1M0 | positive | excluded | type II |
| 51 | 42 | F | 60 | P2N0M0 | negative | type IV | type IV |
| 52 | 40 | F | 59 | P2-3N0MX | N/A | type IV | type IV |
| 53 | 3 | F | 42 | P4N0M0 | positive | type I | type I |
| 54 |  | F | 55 | P3N0M0 | positive | excluded | type I; type IV |
| 55 | 35 | F | 56 | P3N1M0 | positive | type IV | type IIIa; type IV |
| 56 |  | M | 60 | P2N0M0 | positive | excluded | type I |
| 57 | 5 | F | 58 | P2N1M1 | positive | type I | type I |
| 58 | 43 | F | 58 | N/A | negative | type IV | type IV |
| 59 | 39 | F | 31 | P1N0M0 | negative^c^ | type IV | type IV |
| 60 | 31 | F | 30 | N/A | positive | type IIIa | type IIIa |
| 61 | 15 | M | 45 | P2N0M0 | positive | type II | type II |
| 62 | 3 | M | 59 | N/A | positive | excluded | type I |
| 63 | 12 | F | 28 | P2NXM0 | positive | type II | type II |

^a^PNM (Parasitic mass in the liver, involvement of Neighbouring organs, Metastasis) classified according to Kern et al.^2^; ^b^Serology was tested with an enzyme-linked immunosorbent assay (Echinococcus multilocularis – ELISA (Em2-Em18) #9300, Bordier Affinity, Crissier, Switzerland). Tests were performed for diagnostic reasons at different time points of resection/biopsy and determination, and hence not used for further evaluation in this study; ^c^External serological data; EMUC-CT: Echinococcus multilocularis Ulm Classification for computed tomography images; N/A: not applicable
