# Supplementary material for: Combining Computed Tomography and Histology Leads to an Evolutionary Concept of Hepatic Alveolar Echinococcosis
Source: Pathogens. 2020 Aug 4;9(8):634. doi: 10.3390/pathogens9080634 (PMC7459611; doi:10.3390/pathogens9080634)
Supplement: Supplementary file 1 [file pathogens-09-00634-s001.zip › Table S2.docx]

**S2 Table.** Numbers of samples analysed for each type of alveolar echinococcosis lesion (n=45 lesions*)

| **EMUC-CT** | **Samples (%)**  **male-female** | **One slide analysed  (% of EMUC-CT types)** | **Two slides analysed  (% of EMUC-CT types)** |
| --- | --- | --- | --- |
| **All samples** | 45 (100.00%)  12-33 | 10 (22.22%) | 35 (77.78%) |
| **type I** | 11 (24.44%)  6-5 | 0 (0.00%) | 11 (100.00%) |
| **type II** | 11 (24.44%)  2-9 | 0 (0.00%) | 11 (100.00%) |
| **type III** | 10 (22.22%)  2-8 | 1 (10.00%) | 9 (90.00%) |
| **type IV** | 11 (24.44%)  2-9 | 9 (81.82%) | 2 (18.18%) |
| **type V** | 2 (4.44%)  0-2 | 0 (0.00%) | 2 (100.00%) |

*n=44 patients; two samples were analysed from one patient with two different EMUC-CT-type lesions; EMUC-CT: Echinococcus multilocularis Ulm Classification for computed tomography images
