# Supplementary figures and images for: Combining Computed Tomography and Histology Leads to an Evolutionary Concept of Hepatic Alveolar Echinococcosis
Source: Pathogens. 2020 Aug 4;9(8):634. doi: 10.3390/pathogens9080634 (PMC7459611; doi:10.3390/pathogens9080634)

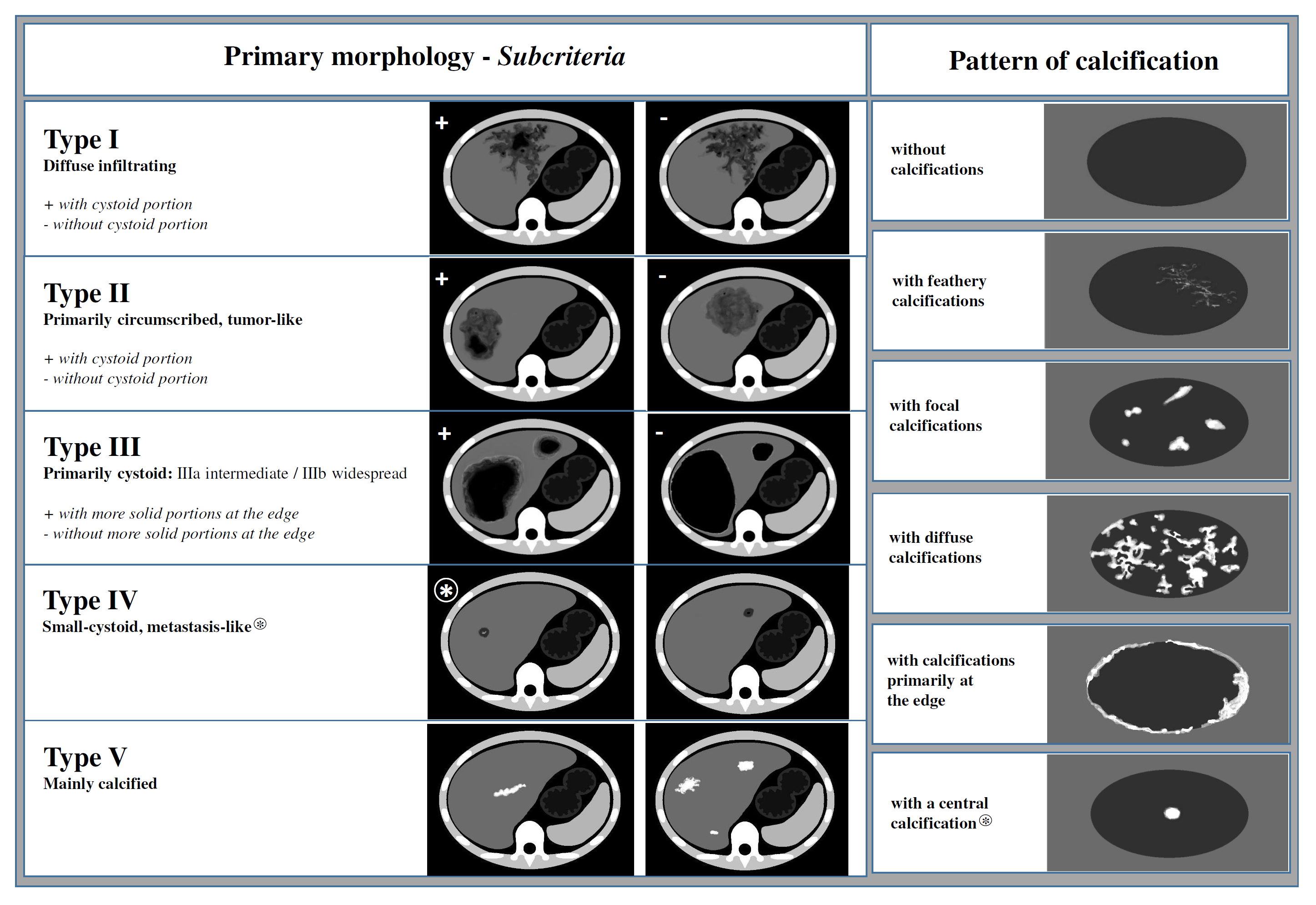

Supplement: Supplementary file 1 [file pathogens-09-00634-s001.zip › Figure S1.tif]

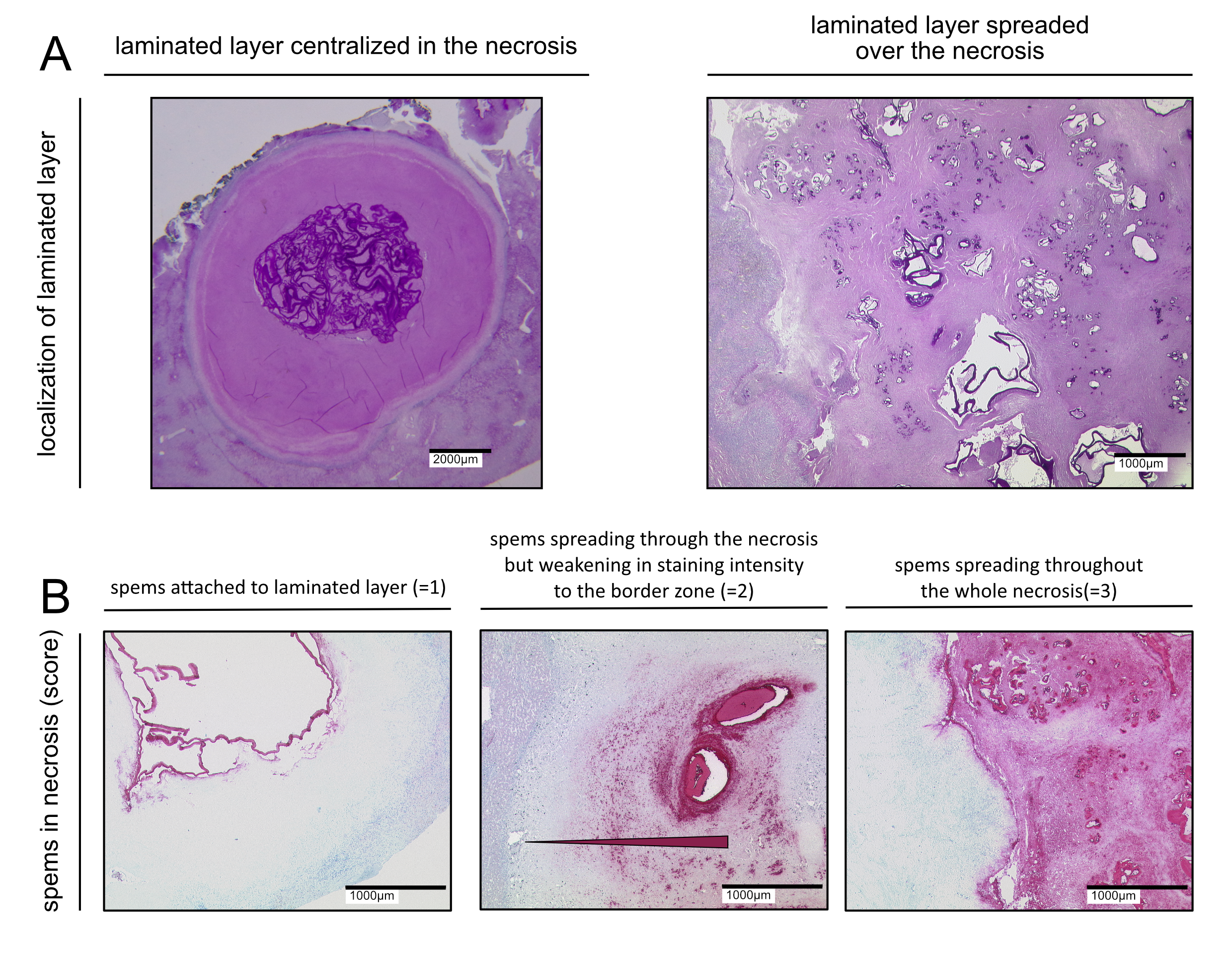

Supplement: Supplementary file 1 [file pathogens-09-00634-s001.zip › Figure S2.tif]
